# Intentional communication between wild bonnet macaques and humans

**DOI:** 10.1038/s41598-018-22928-z

**Published:** 2018-04-12

**Authors:** Adwait Deshpande, Shreejata Gupta, Anindya Sinha

**Affiliations:** 1 Consciousness Studies Programme, National Institute of Advanced Studies, Bangalore, India; 2 Animal Behaviour and Cognition Programme, National Institute of Advanced Studies, Bangalore, India; 3 Primate Programme, Nature Conservation Foundation, Mysore, India; 4 Centre for Neuroscience, Indian Institute of Science, Bangalore, India; 5 Dhole’s Den Research Foundation, Bandipur National Park, Karnataka, India

## Abstract

Comparative studies of nonhuman communication systems could provide insights into the origins and evolution of a distinct dimension of human language: intentionality. Recent studies have provided evidence for intentional communication in different species but generally in captive settings. We report here a novel behaviour of food requesting from humans displayed by wild bonnet macaques *Macaca radiata*, an Old World cercopithecine primate, in the Bandipur National Park of southern India. Using both natural observations and field experiments, we examined four different behavioural components—coo-calls, hand-extension gesture, orientation, and monitoring behaviour—of food requesting for their conformity with the established criteria of intentional communication. Our results suggest that food requesting by bonnet macaques is potentially an intentionally produced behavioural strategy as all the food requesting behaviours except coo-calls qualify the criteria for intentionality. We comment on plausible hypotheses for the origin and spread of this novel behavioural strategy in the study macaque population and speculate that the cognitive precursors for language production may be manifest in the usage of combination of signals of different modalities in communication, which could have emerged in simians earlier than in the anthropoid apes.

## Introduction

Organised human language is perhaps one of the most important behaviours that distinguish human beings from all other species^1,2^, with intentionality lying at the core of this communication system^3–6^. The biological origin and evolution of this crucial dimension of human language, however, remains underexplored but could benefit from comparative studies of the communication systems of our closest living relatives, nonhuman primates (henceforth, primates).

Intentional communication in humans requires an understanding of the mental states of articulators, which, in turn, requires complex cognitive capacities^7,8^. Subsequently, intentionality in animal communication systems was operationalised through different orders of ‘intentionality’^9–11^. Zero-order intentionality, for example, is merely reflexive communication, not involving any sophisticated cognitive processes. This then proceeds to higher orders of intentionality, which involves some recognition of one’s own mental states as well as those of audience, culminating in the capacity of the actor to successfully communicate its own intentions and goals to the audience, the characteristic feature of higher-order intentionality^10–13^. Although this approach is practically helpful to identify intentionality in human communication, recent studies have suggested that mental-state attribution may not be a necessary criterion to typify intentional communication, particularly in non-humans^13^. This has given rise to various general behavioural criteria to qualify a communicative signal to be intentional. These criteria include (1) the social use of the communicative act, as indicated by the signal being directed to particular recipients, modified by various factors, such as the presence or composition of the attendant audience; (2) sensitivity of the signaller to the attentional states of the recipients; (3) manipulation of the attentional states of recipients to attract attention, particularly when a mutual attention state between the signaller and recipient is absent or the signaller moves itself into the line of view of a recipient; (4) monitoring the responses of the audience and (5) persistence in the production and/or elaboration of the signal until the desired communicative goal is met^4,14,15^ (but see ref.^13^ for a recently proposed, simpler framework for intentional communication in animals).

The use of these criteria is largely restricted to the great ape communication systems and several studies have revealed that, among various modalities used in primate communication, gestures represent intentional signals^3,16–24^. Investigations into such intentional gestures have, however, mostly remained restricted to great ape communication systems. A few recent studies on captive red-capped mangabeys *Cercocebus torquatus*^25^, olive baboons *Papio anubis*^26–28^, Tonkean macaques *Macaca tonkeana*^29^, and rhesus macaques *Macaca mulatta*^30^ have revealed the capacity of these monkeys to intentionally direct learnt gestures towards humans in certain specific contexts, akin to that shown by captive chimpanzees *Pan troglodytes*^17,21,31^, gorillas *Gorilla gorilla*^32,33^ and rehabilitated orangutans *Pongo pygmaeus*^34^. To the best of our knowledge, the only documentation of intentional gestural communication directed by monkeys towards conspecific individuals in the wild is that from our long-term observations of bonnet macaques *M*. *radiata* in the Bandipur National Park of southern India^35^.

In contrast to gestures, intentionality has been rarely implicated in primate vocalisations; primate calls have generally been considered to be reflexive and nonflexible^6,36–38^. Nevertheless, recent studies on wild chimpanzees, which used the same criteria for intentionality, showed that certain intentional alarm calls may take into account the attentional states of the audience, contain more complex information than previously thought, including the presence and levels of threat from specific predators, and attract more audience attention than those produced randomly^12,39^. Recent studies on different primate species, both in captivity and in the wild, however, have revealed an inherent flexibility in certain call-types^12,40–57^, questioning the extant notion of primate vocalisations being largely fixed and non-intentional.

An important communicative interaction, characterised by its underlying intentionality, is that of requesting for food, directed by certain captive primates—including chimpanzees^19,21^, mangabeys^25^, macaques^29,30^, and baboons^26–28,58^—towards their human caregivers. This experimental paradigm has, however, faced criticism as being a possible artefact of captivity^6,59^. Our long-term studies on wild bonnet macaques have, however, revealed food-requesting behaviour towards humans to be an intrinsic component of the behavioural repertoire of several free-ranging individuals in two independent populations of this species, reflecting contexts and situations similar to that in the experiments conducted in captivity^60,61^. The bonnet macaque is an Old World cercopithecine primate, endemic to peninsular India and extensively distributed across a wide range of habitats, possibly due to its exceptional ecological flexibility^62,63^. The presence of an elaborate and flexible behavioural repertoire^64,65^, complex social interactions mediated by cognitive decision-making^66^, innovations in communication behaviour^67^ and varied behavioural traditions in different populations of the species^60^ provide further evidence of the phenotypic plasticity that characterises this primate.

**Figure 1:**
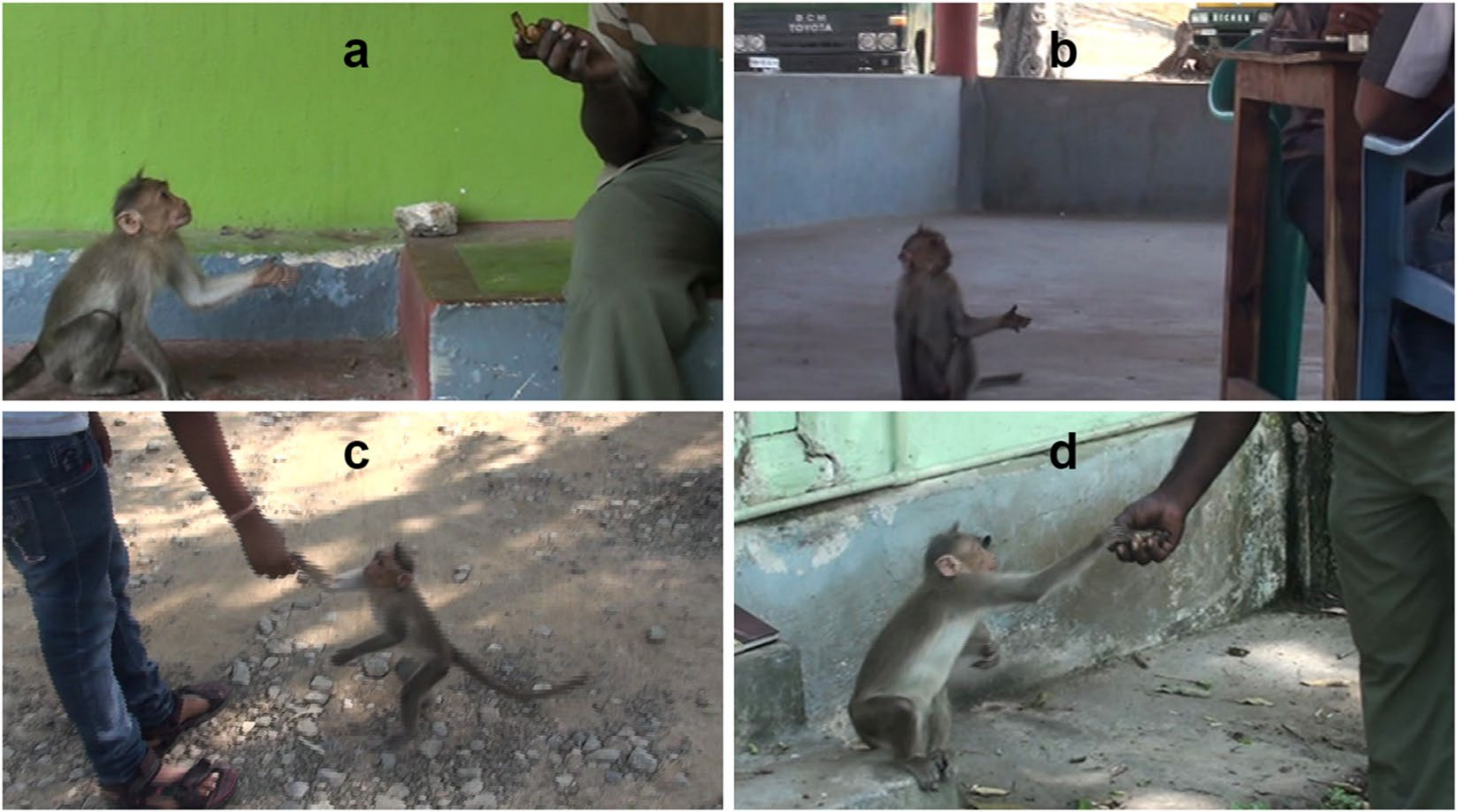
Hand-extension gesture, displayed by juvenile bonnet macaques during food-requesting events (**a**,**b**), as distinguished from the act of reaching out for food (**c**,**d**).

Our preliminary observations revealed four distinct behaviours— coo call, hand-extension gesture (Fig. 1), orientation and monitoring behaviour (Table 1 and see supplementary material)—to be displayed by bonnet macaques during food requesting from humans. Coo-calls have traditionally been observed to serve as a signal to maintain auditory contact or group cohesion in Japanese and rhesus macaques^68–70^, a function that we too recorded in our study troops. The use of the coo-call by bonnet macaques, when requesting food from humans, described in the present study, however, appeared to be an entirely novel application of a well-known macaque vocal signal in this species, especially when accompanied by the display of a novel hand-extension gesture.

In this study, we characterise the structural components and the functional organisation of food-requesting behaviour, a novel interaction observed between wild bonnet macaques of our study group and humans. We approached this investigation with natural observations of food-requesting events and complementary field experiments. We explored the effects of the presence of food and varying attentional states of the humans, from whom the macaques were requesting food, on the four components of the observed food-requesting behaviour. We also tested these behavioural components for compliance with established behavioural criteria of intentional communication, listed above.

## Results

The study bonnet macaques produced coo-call vocalisations invariably during natural observations and experimental trials of food-requesting events while a novel gesture, the hand-extension, was performed, on average, on half the occasions (Table 2). Although the two signals—coo call and hand-extension gesture—were often displayed within the same food-requesting event or experimental trial, we did not examine the precise temporal relationship between these signals of different modalities. Of the 86 food-requesting events recorded during natural observations, the subject macaques received the requested food item in 33 instances (38.3%), out which on five occasions subject macaques acquired food by stealing it when left unattended by the target human. Human targets chased away subject macaques on the other remaining 53 (61.7%) occasions. The study individuals did not display any component of the food-requesting behaviour after receiving the food item from the respective human target during any of the requesting events.

**Table 1:** Different behavioural components of food-requesting events displayed by juvenile macaques.

Behavioural component	Description/Definition
Coo-call	Low-frequency vocalisation with minimal frequency modulation [Supplementary Figure S2]
Hand-extension gesture	Extension of either of the hands, with an open palm (but occasionally with fingers clenched) towards the human recipient Gesture distinguishable from the act of reaching out for food by the holding of the arm below the shoulder-level, with the palm facing upward, for a relatively prolonged duration of time (Fig. 1)
Monitoring behaviour	Head of the subject macaque completely orientated towards the human recipient, with direct visual attention being paid to the recipient, being considered as visual monitoring of the recipient and/or the food item held by the recipient, at a maximum distance of ≈8 m from the recipient [Supplementary Figure S3]
Orientation behaviour	Body movements or locomotor behaviour (change in position) displayed by a subject macaque, resulting in its positioning itself in the visual field of the recipient’s line of vision, at a maximum distance of 8 m from the recipient [Supplementary Figure S4]
Mutual visual attention	The head of a subject macaque being orientated towards a human recipient and the latter’s body and head also orientated towards the former, with the two individuals apparent gazing at one another, at a maximum distance of 8 m between them

**Table 2:** Characteristics of the food-requesting events displayed by juvenile bonnet macaques during natural observations and field experiments.

	Sample size	Duration of event (min) Mean ± SE, Range	Rates of coo-calls (acts/min) Mean ± SE Range	Rates of hand-extension gestures (acts/min) Mean ± SE Range	Orientation behaviour (proportion of time spent) Mean ± SE Range	Monitoring behaviour (proportion of time spent) Mean ± SE Range
Natural observations	86 events, 18 individual macaques	2.02 ± 0.19 0.18–8.50	3.85 ± 0.30 1–13	0.33 ± 0.07 0–2.90	0.14 ± 0.01 0.01–0.74	0.39 ± 0.02 0.02–0.98
Field experimental trials	24 trials, 4 individual macaques	40 sec (0.66 min)	4.70 ± 0.50 1–15.15	1.30 ± 0.34 0–12.12	0.14 ± 0.03 0–0.77	0.57 ± 0.02 0.02–0.93
Field control trials	24 trials, 4 individual macaques	40 sec (0.66 min)	1.00 ± 0.35 0–9.00	0	0	0.16 ± 0.01 0.01–0.52

### Effect of the presence of food on food-requesting behaviour: Field experiments

**Figure 2:**
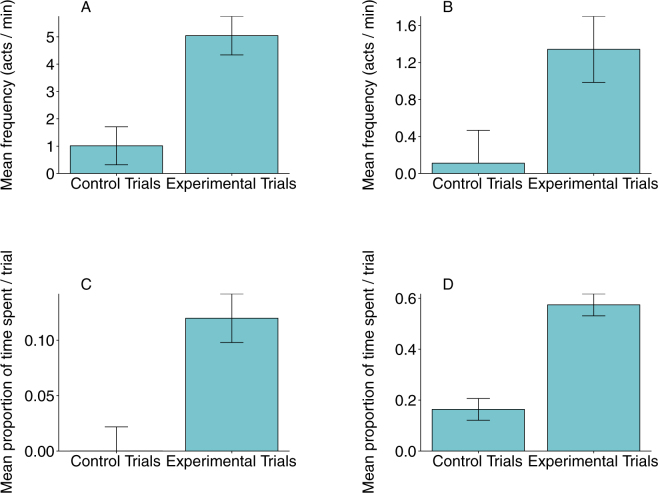
Mean (±SE) frequency of (**A**) coo-call (n = 48, p < 0.001) and (**B**) hand-extension gesture displayed (n = 48, p < 0.001) and proportion of time spent in (**C**) orientation behaviour (n = 48, p < 0.001) and (**D**) monitoring behaviour (n = 48, p < 0.001) by the subject macaques as a function of the presence (experimental trials) or absence (control trials) of food during the field experiments.

The subject macaques exhibited all the four behavioural components at significantly higher levels in the experimental (with food) trials, as compared to the control (without food) trials (Linear mixed-effect models; Coo-call: X^2^_1_ = 76.01, n = 48, p < 0.0001; Hand-extension gesture: X^2^_1_ = 45.05, n = 48, p < 0.0001; Orientation behaviour: X^2^_1_ = 45.28, n = 48, p < 0.0001; Monitoring behaviour: X^2^_1_ = 108.11, n = 48, p < 0.0001; Fig. 2).

### Effect of the attentional states of humans on food-requesting behaviour: Natural observations and field experiments

The rate of hand-extension gestures was significantly higher during the ‘direct attention’ state (Wilcoxon’s matched-pairs signed-ranks test, two-tailed; Natural observations: Z = 3.89, V = 207, n = 86, p = 0.0001, r = 0.41; Experimental trials: Z = 3.18, V = 114, n = 24, p = 0.001, r = 0.64) while the proportion of adjustment of orientation behaviour was higher during the ‘no attention’ state (Natural observations: Z = 0.77, V = 4, n = 86, p < 0.0001, r = 0.08; Experimental trials: Z = 3.61, V = 2, n = 24, p = 0.0003, r = 0.73). There were no significant differences in the rate of coo-calls (Natural observations: Z = 1.07, V = 1544, n = 86, p = 0.28, r = 0.11; Experimental trials: Z = 0.89, V = 65, n = 24, p = 0.30, r = 0.18) and duration of monitoring behaviour (Natural observations: Z = 2.70, V = 1210, n = 86, p = 0.34, r = 0.29; Experimental trials: Z = 0.69, V = 72.5, n = 24, p = 0.48, r = 0.14) displayed by the subject macaques in response to the attentional state of the human interactants.

**Figure 3:**
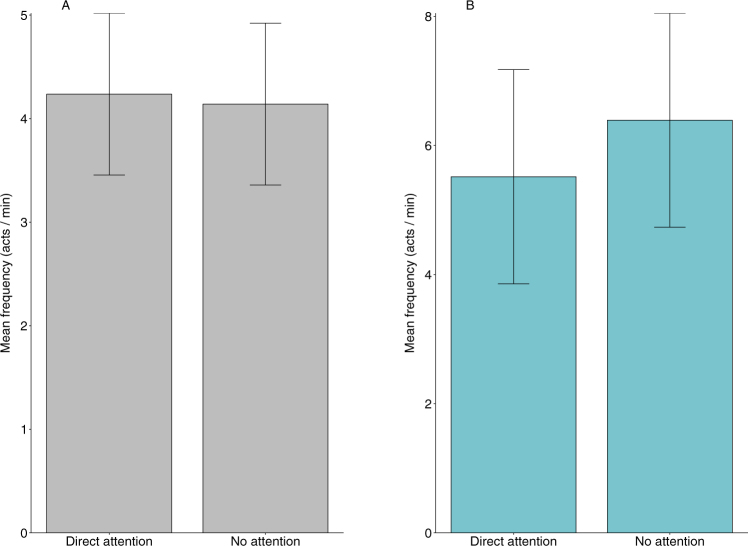
Mean (±SE) frequency of coo-call displayed by the subject macaques as a function of the two attentional states of the (**A**) target humans during natural observations (n = 86, p = 0.88) and the (**B**) human subjects during the experimental trials (n = 24, p = 0.30).

Linear mixed-effect models of the observational and experimental data revealed that the attentional states of the target humans (direct and no attention) did not explain the rate of coo-calls produced by the food-requesting macaques during natural observations (X^2^_1_ = 0.02, n = 86, p = 0.88) or of the human subjects during the experimental trials (X^2^_1_ = 1.06, n = 24, p = 0.30; Fig. 3).

**Figure 4:**
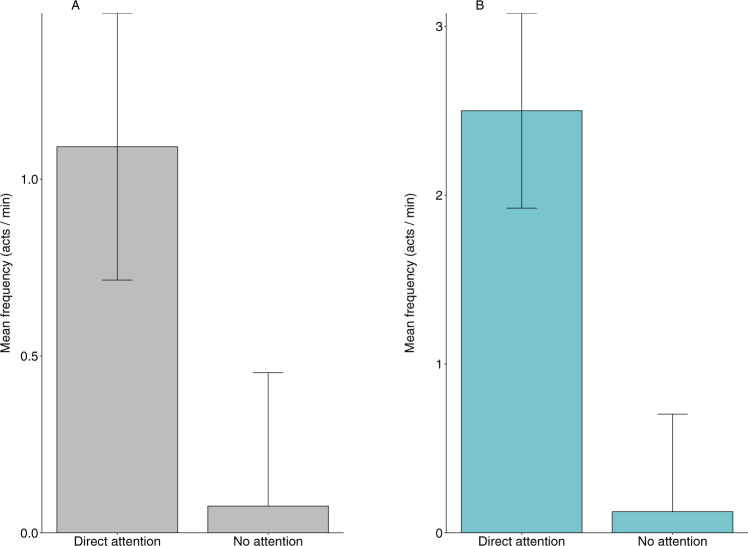
Mean (±SE) frequency of hand-extension gesture displayed by the subject macaques as a function of the two attentional states of the (**A**) target humans during natural observations (n = 86, p < 0.001) and the (**B**) human subjects during the experimental trials (n = 24, p < 0.001).

The food-requesting macaques, however, produced the hand-extension gesture at higher frequencies only when there was mutual visual attention between the human subjects and the macaques, both during natural observations (X^2^_1_ = 14.34, n = 86, p < 0.001) and in the experimental trials (X^2^_1_ = 15.19, n = 24, p < 0.001; Fig. 4).

**Figure 5:**
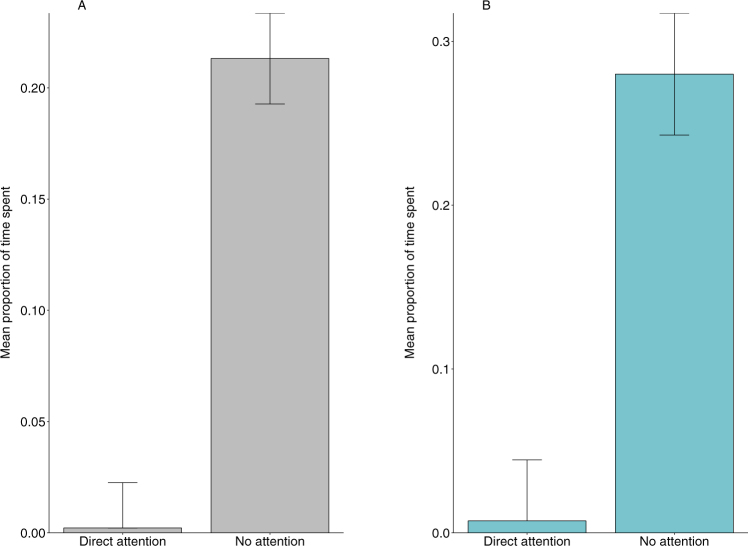
Mean (±SE) proportion of time spent in orientation behaviour by the subject macaques as a function of the two attentional states of the (**A**) target humans during natural observations (n = 86, p < 0.001) and the (**B**) human subjects during the experimental trials (n = 24, p < 0.001).

The duration of orientation behaviour, displayed by the study macaques, was significantly higher when the target humans were not attending to the subject macaques, again both during natural observations (X^2^_1_ = 67.92, n = 86, p < 0.001) and in the experimental trials (X^2^_1_ = 22.74, n = 24, p < 0.001; Fig. 5).

**Figure 6:**
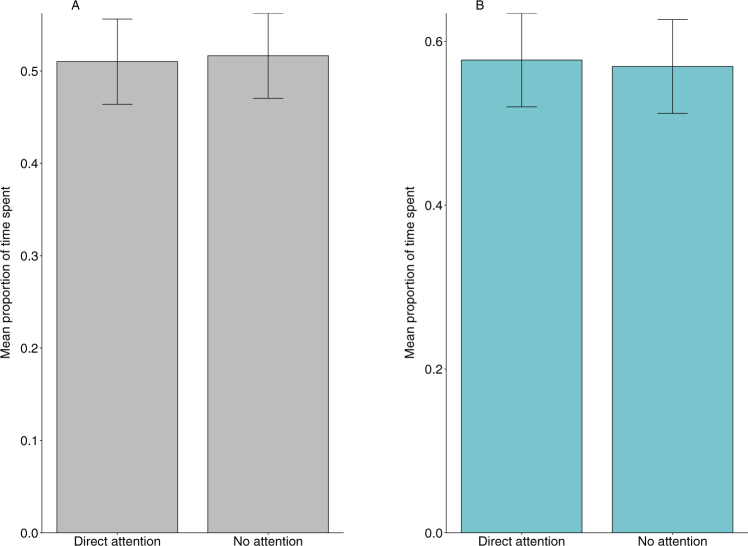
Mean (±SE) proportion of time spent in monitoring behaviour by the subject macaques as a function of the two attentional states of the (**A**) target humans during natural observations (n = 86, p = 0.75) and the (**B**) human subjects during the experimental trials (n = 24, p = 0.63).

Finally, there were no significant differences in the performance of monitoring behaviour by the macaques in response to human attentional state, either during natural observations (X^2^_1_ = 0.09, n = 86, p = 0.75) or in the experimental trials (X^2^_1_ = 0.22, n = 24, p = 0.63; Fig. 6).

## Discussion

We describe here a novel communication system: food-requesting behaviour in wild, untrained monkeys, interacting with unfamiliar human beings. This behaviour appears to have emerged in at least two natural populations of bonnet macaques, an Old World cercopithecine primate species, in both of which it has already been established as a distinct behavioural tradition^60,61^. This signalling system involves, on occasion, signals of different modalities; specifically, a combination of vocal and two visual signals and remarkably, it was always exclusively directed towards humans. Although the macaques used communicative signals in different modalities within the same food-requesting event, we did not examine the temporal relationship between any two consecutive signals across modalities and hence, could not establish whether this communication system is multimodal in nature.

Communication signals in non-ape primates have rarely been established to be intentional and referential, especially in the wild^35^ (but see^25,26,29,30^ for studies in captivity). We tested for the compatibility of the behavioural components of food-requesting behaviour, as displayed by the study macaques, with the key criteria laid down to qualify a certain communication signal as being intentional in nature. First, our natural observations indicated that two key behavioural components—the coo-call and hand-extension gesture—were almost invariably used in contexts wherein the macaques interacted with humans. While the hand-extension gesture was never displayed in any other situation, coo-calls were produced in one other social context, when juvenile bonnet macaques were separated from their natal troops and unable to detect them. In the field experiments conducted during this study, the subject macaques performed all the behavioural components at significantly higher levels only when human subjects carried food during the trials (experimental trials), as compared to without food trials (control trials). This suggests that these communicative behaviours were being directed only towards a particular recipient, a human holding a food item. These signals thus qualify the first criterion of intentional communication, which proposes the presence of an appropriate recipient towards whom a signal needs to be directed^3,12,20,44,71^.

The hand-extension behaviour was displayed by the macaques only when there was a mutual visual-attention state established with the human recipient, as measured in terms of the two subjects exhibiting complete body orientation towards one another, as recorded during the natural observations and experimental trials. This establishes the credentials of this behaviour being intentional, as the second criterion of intentionality demands that the signaller be sensitive to the attentional state of the recipient. Visual gestures in primates have been defined to be movements of the limbs or head and body that are directed towards a recipient and that are goal-directed or intentional, and mechanically ineffective actions^72^. In accordance with this definition, we here report previously unreported “visual gesture” of hand-extension used in a specific communicative context for this population of bonnet macaques which was absent from gestural repertoire of this species^35^.

Coo-calls have traditionally been observed in different macaque species and preliminary used as a contact call that serves as a signal to maintain group cohesion^68–70^. During our focal animal sampling, we too recorded usage of coo-calls on eight occasions when juvenile macaques were separated from their natal troops. To our knowledge, this is the first record of wild bonnet macaques using the coo-calls, in a novel context of requesting food from humans. Our observations of the involvement of this call in the context of food acquisition by bonnet macaques may, however, not be entirely unprecedented. Rhesus macaques^73,74^ and Japanese macaques^75^, for example, could be trained to emit coo-calls while requesting food from humans but only under captive conditions. It is significant to note, in this connection, that two Japanese macaque individuals spontaneously produced coo-calls when taught to use a tool to retrieve food from an otherwise inaccessible source^75^. None of the studies, however, examined the actual function of the coo-call in those particular contexts.

We hypothesised that a potential function of the bonnet macaque coo-call in the context of food requesting would be to attract the visual attention of the human subject during the interaction. If true, we would predict that the macaques would produce this call at relatively higher rates in the absence of mutual visual attention—a condition that we tested for, both in our natural observations as well as in the field experiments. Our analysis of the coo-calls produced by the study macaques, however, revealed no significant differences in the rates of their production in relation to the attentional state of the human recipients, either during natural observations or in the field experiments. This would, therefore, tentatively argue against a possible role of the coo-calls to serve as a vocalisation to attract the visual attentional state of the humans, from whom the macaques were requesting food.

Alternatively, the call could be used to express the caller’s communicative intent to acquire food from humans; it could also serve the purpose of maintaining persistent contact with the human recipients. It has, in fact, been suggested that a signal could simultaneously function to express the internal motivational states of the signaller as well as respond to the external events experienced by the signaller^5,76,77^. We must, however, reiterate that it may be empirically difficult to distinguish between the latter possible functions of the coo-call. Additionally, it is entirely possible that certain other factors, such as partial occlusion of the food item, a specific position of the food item or some other unaccounted variable could explain the usage of coo-calls during food requesting. These possibilities, however, remain unexplored in our present study.

The study macaques displayed orientation behaviour both during natural observations and the field experiments when there was no mutual visual attention state with the humans involved. The macaques performed this behaviour consistently across food-requesting events and persistently within each event in a way that suggested that they were attempting to orient themselves to the visual field of the human recipients. This indicates that the bonnet macaque’s orientation behaviour functions to attract the attentional state of human interactants in this particular context by the subject macaque moving into the line of vision of the humans, illustrating the third criterion for intentional communication, which suggests that a signaller would orient itself with the line of view of a recipient.

The fourth criterion of intentionality requires continuous monitoring of the recipient by a signaller. This was met by the monitoring behaviour shown by the study macaques during natural observations or in experimental trials whenever food was present, regardless of the attentional state of the human interactants.

The final key criterion of intentional communication refers to the persistence and elaboration of a signal. Although debated, persistence is generally displayed when a goal-directed behaviour is expressed repeatedly or a functionally similar signal is displayed until the desired goal is achieved^6,13^. The study macaques ceased to display the behavioural components of food requesting immediately after they received the food item from the human target, which, we argue, marked the achievement of their intended goal. We suggest that, in future studies, experimental manipulation of goals could possibly be used to quantify persistence in a more concrete, objective manner^13^.

Another aspect of persistent behaviour—that of elaboration—allows for the enhancement of a signal in order to achieve the same goal and appears to be a crucial factor in intentional communication^19,78^. Traditionally, elaboration has been observed and discussed in the light of unimodal communication and involves instances when the signaller may have switched from the principal signal to another one in a different modality^79^. In the present study, we propose that the bonnet macaques have already elaborated on their signal by developing a signalling strategy that involves at least two visual signals (hand-extension gesture and orientation behaviour) and coo-calls. We further argue that it may be a difficult exercise to determine the principal signal in this communication system. We, therefore, suggest that the study macaques have possibly achieved the function of signal enhancement through a use of multiple visual or vocal signals.

Food requesting by the bonnet macaques thus appears to be potentially an intentionally produced behavioural strategy, as all its behavioural components except coo-calls conform to all the key multiple criteria of intentionality, considered simultaneously^12,80^. The argument against this complex behaviour being a mere response to the stimuli of food presence is bolstered by our observations that the simple sight of food items, available naturally or held by conspecific individuals, neither elicited a coo-call nor a hand-extension gesture in any situation. Moreover, in five of the 86 food-requesting events that we recorded, the subject macaques changed their strategy of acquiring food from requesting to opportunistically snatching the food item, when it was left unattended. This too indicated that the macaques displayed the food-requesting behaviour with the intention of acquiring the desired food item.

The food-requesting behaviour displayed by our study bonnet macaques involves communication signals that are being used intentionally. What is novel, however, is that these signals are being employed by the macaques through frequent interactions with humans in the wild; the performance of these behaviours has never involved any active training by caretakers, unlike that reported in studies of intentional gesturing by captive monkeys^25,26,29,30^. It is important to speculate on the possible origins of this novel behavioural strategy in particular populations of bonnet macaques. In general, bonnet macaques rarely actively share food with conspecifics and are more likely to aggressively defend food resources from others^6,59,81,82^. Adult bonnet macaques in Bandipur rarely displayed the food-requesting behaviour while interacting with humans holding food items. They either opportunistically snatched the food items away or aggressively threatened their human targets, the latter often being an effective strategy to induce their targets to throw away the food (AD and AS pers. obs.).

Juvenile macaques, which have begun to forage independently, however, may have to depend on alternative foraging strategies such as food requesting, particularly given their small body size and general fear of humans. All the 18 juveniles observed to display food-requesting behaviour in Bandipur were between the ages of two to four years. The additional ecological opportunity to request food from heterospecific humans could have potentially given rise to the co-option of the plaintive coo-call, generally produced by individuals of this species when separated from their troops, and the development of a new gesture, that of hand extension, by juvenile macaques in a novel context. It is entirely possible that, initially, certain individuals could have acquired the hand-extension gesture from simply reaching out with their forelimbs for the food held by humans. This behavioural act could have then become ritualised through frequent interactions with humans and may have either been reinforced when positively rewarded and then spread by social learning mechanisms, such as social facilitation or emulation within the population. Many vertebrate species are, in fact, known to rely on social learning to develop and acquire novel and/or complex foraging techniques^83–89^. More focused studies are required in the future to test these hypotheses in this population of bonnet macaques.

In conclusion, our study is the first endeavour to explore intentionality underlying complex vocal and gestural communication between a wild, non-ape primate species and humans in an entirely natural context, thus providing support to similar studies conducted in captive settings^25,27–30^. It also highlights an inherent capacity of behavioural flexibility displayed by a non-human species in using communication signals of different modalities to achieve an intended goal. Our present study thus provides strong supporting evidence toward inherent capacities of intentional communication in macaques, thus encouraging us to think that the precursors of intentional behaviour are possibly evolutionarily older than previously thought, even preceding that in the anthropoid apes. This exploration also tempts us to speculate that the cognitive precursors for language production may possibly be manifest in the usage of a combination of signals of different modalities to communicate particular intent^90^. Our preliminary explorations of such potentially evolutionarily conserved signalling could also aid future studies in investigating the origins and cognitive roots of human language. More specifically, we establish once again the inherent capacity of bonnet macaques, an Old World cercopithecine species, to develop and employ novel behavioural strategies and communicate their intent to members of another species in an unusual context, reaffirming the remarkable behavioural flexibility and cognitive abilities of this species^60,63,65–67^.

## Methods

### Study site and troops

This study was conducted in the Bandipur National Park (11.67°N, 76.63°S), situated in the state of Karnataka in southern India. The Park extends over 874 km^2^, with a range of various habitats including dry deciduous forests, moist deciduous forests and scrublands.

We studied two neighbouring, human-habituated troops of bonnet macaques, comprising 60 and 32 individuals respectively, from December 2014 to March 2015. The home ranges of the troops largely overlapped with one another and encompassed several locations frequented by tourists visiting the Park, providing opportunities for the macaques to interact with them and acquire provisioned food [Supplementary Figure S1].

### Natural observations

We conducted a preliminary survey to identify the locations, within the home ranges of the troops, where the study individuals interacted with people or juvenile macaques displayed food-requesting behaviour and determined Site 1 to display the highest frequency of such interactions [Supplementary Figure S1]. All the naturally occurring food-requesting events that were observed and analysed in this study were located at this site and involved a subject juvenile macaque and a target human of either sex for both species. Individual macaques were identified based on their morphological features. All-occurrence sampling was used to record each event, with its constituent interactions^91^.

A food-requesting event typically consisted of four distinct behavioural components—coo-calls, hand-extension gestures, orientation- and monitoring behaviours—performed by the subject juvenile macaque (Table 1), at least one of which was performed during each event. We also classified the visual attentional state of the human target in each event into the two following categories.

(A) Direct attention: The body- and/or head orientation of the human target is towards the subject macaque.
(B) No attention: The human subject did not make eye contact with and maintained a head and body orientation at least 90° away from the subject macaque.

A food-requesting event was considered to have been initiated when an individual macaque arrived at Site 1 and produced the first coo-call or hand extension gesture of the event in the presence of a human recipient. We considered the event to have ended when the subject macaque successfully acquired a food item from the human target, was chased away by the human target or other bystanders, or voluntarily left the site without obtaining the food item. We followed the subject macaque for the next ten minutes after each food-requesting event and recorded all the behaviours displayed. The number of human bystanders present during each event and the total duration of the event were also recorded.

The entire food-requesting event, with its constituent behaviours, was video-recorded using a Sony HDR-SR10E digital video camera (Sony, Tokyo, Japan). A total of 140 food-requesting events were recorded over a period of 78 days (mean ± SE of 1.7 ± 0.28 per day), out of which 86 events were chosen for the final analyses, based on their overall audio-video quality, ensuring that both subject macaque and the human recipient of the interaction were clearly visible throughout the clip [Supplementary Video S1]. We also ascertained that the food items held by the humans were completely visible and were in the line of vision of the subject macaques in all the 86 events.

We used focal animal sampling, of 15-min duration each, on randomly selected individual macaques, without replacement, from both study troops to gather information on the usage of coo-calls and the hand-extension gesture in different contexts^91^. We conducted a total of 432 focal animal samples on 37 focal adult, sub-adult and juvenile macaques of both sexes, amounting to 108 h of observation. All behavioural events, including coo-calling and foraging acts, were recorded during this sampling, following an unpublished ethogram of the species (Sinha, pers. obs.).

### Field experiments

We also conducted experimental trials on four selected juvenile male macaque subjects, ranging from 1.5 to 3 years of age, belonging to either study troop. These individuals were selected based on their (a) relatively higher tendency of approaching and interacting with humans, and (b) relatively higher frequencies of displaying food-requesting behaviour during observations. We also employed four trained male human volunteers as subjects for these field experiments, with each individual being selected randomly for each trial.

During the experiments, we controlled for two visual attentional states of the human subject—direct attention and no attention—similar to those categorised and analysed in the natural observations, as described below.

(A) Direct attention: The human subject maintained continuous eye contact and complete body orientation with the subject macaque.
(B) No attention: The human subject did not make eye contact with and maintained a body and head orientation of 180 degrees away from the subject macaque, turning away, if necessary, in cases when the macaque moved to confront the human subject.

We designed experimental and control trials to test for the effect of these attentional states on the food-requesting behaviour displayed by the subject macaques. In each experimental trial, the human subject, dressed appropriately to simulate a visiting tourist, approached a macaque subject to an approximate distance of 3 m, holding a food item in one hand away from the torso but easily visible, thus initiating the trial. Once initiated, the human subject maintained either of the two attentional states, chosen randomly for 20 sec and then switched to the other attentional state, which he maintained for another 20 sec. The trial was terminated after 40 sec with the human subject walking speedily away from the macaque, without any further eye contact and without providing the food item to the macaque [Supplementary Video S2]. During all the experimental trials, the food item held by the experimenter was always at least partially visible to the subject macaque.

We also conducted control, or without-food trials, which were identical to the experimental trials except that the human subjects did not carry any food item during the trial. Every trial, experimental or control, was video-recorded at a distance of 5–10 m from the event. All trials were conducted only when the subject macaques were foraging or sitting away from the parent troop and no other individual macaques were in the vicinity. We conducted a total of 12 trials on each of the four subject macaques—six experimental and six controls—amounting to a total of 48 trials. Care was taken to ensure that each subject macaque was tested only once on a particular day and any trial on the same subject was conducted with a minimal gap of 36 h. The four human subjects were used comparably in the 48 trials.

### Ethics statement

Permission to conduct the natural observations and field experiments on the study bonnet macaque troops were obtained from the Conservator of Forests and Director of the Bandipur National Park. All video recordings of the natural human-macaque interactions were made only after permission was received from the respective people involved. All the human subjects participated voluntarily in the field experiments. We obtained the informed consent of all the human subjects regarding the use of videos and images for data analyses and publishing. All methods were performed in accordance with the relevant guidelines and regulations. The Research Ethics Committee of our host institution, the National Institute of Advanced Studies in Bangalore, approved all the protocols for the natural observations and field experiments.

### Behavioural video coding

The video recordings were played at 25 frames/sec and coded by AD using BORIS Version 2.2^92^. For analysis, we focused mainly on the four behavioural components, displayed by the subject macaques in the natural observations or the subject macaques in the field experiments (Table 1). We calculated the rates of occurrence of the coo-call and hand-extension gesture, both being point events, per unit time while the orientation- and monitoring behaviours were measured in terms of the proportion of observed time spent in these behavioural states during the food-requesting events. The performance of these four behaviours was monitored in response to the two attentional states of the target humans in the natural observations and of the human subjects in the field experiments. The total duration of time spent by the target humans in the two attentional states during the food-requesting events was measured from the video recordings of the natural observations.

In order to assess inter-observer reliability in video coding, approximately 20% of the video data were analysed by a human volunteer, who was blind towards the objectives of this study. We conducted separate analyses for coo-calls and the hand extension gesture, yielding a mean Cohen’s kappa coefficient of 0.76 (range = 0.73–0.79)^93^. We also calculated Spearman’s rho between two coders for the proportion of time spent in orientation and monitoring behaviour and found strong positive correlations between them in each case (Orientation behaviour: n = 80, r_s_ = 0.823, p = 0.002; Monitoring behaviour: n = 80, r_s_ = 0.865, p = 0.001).

### Statistical analyses

We used linear mixed-effect models (LME) for the four continuous response variables, rates of coo-calls and hand-extension gestures, and the proportion of time spent in orientation- and monitoring behaviours, but only when the quantile-quantile plots of these variables were in accordance with normal distribution; else, we scaled and centered the variables using a “scale” function in R to fit the normal distribution^94^.

We first tested for the effect of the presence of food on the four behavioural components displayed by the subject macaques in the experimental (with food) and control (without food) field trials (n = 48). We ran four LMEs, using the lme4 package of R^95^ for these four response variables respectively, with the presence of food being considered as an explanatory variable in binary form and with the subject macaques and humans being included as random effects^94^.

To test for differences in the performance of the four behavioural components in response to the two attentional states of the human subjects in the experimental trials, we conducted two-tailed Wilcoxon’s matched-pairs signed-ranks tests for the 24 experimental (with food) trials and 86 natural observations of food-requesting events^96^. Furthermore, to test for the effect of attentional states on coo-calls, the hand-extension gesture, and orientation and monitoring behaviour, we used LMEs to analyse the data from both natural observations and the field experiments. For the natural observations (n = 86), we compared the effect of the predictor variable—the attentional state of the human targets—on the four response variables, while the identities of the subject macaques, identity of the requesting event, number of bystanders present, and duration of the food-requesting event were considered as random effects. Similar models were run for the field experimental trials (n = 24), in which the identities of the subject macaques, humans (experimenters), experimental trial and trial order were added as random effects. We compared all the LMEs with their respective null models to test for their significance. All statistical analyses were performed in R, Version 3.3.1, with levels of significance tested at p = 0.05^97^.

## Electronic supplementary material

Supplementary Materials

Video S1

Video S2
