# Supplementary material for: Intentional communication between wild bonnet macaques and humans
Source: Sci Rep. 2018 Apr 12;8:5147. doi: 10.1038/s41598-018-22928-z (PMC5897542; doi:10.1038/s41598-018-22928-z)
Supplement: Supplementary file 1 — Supplementary Materials [file 41598_2018_22928_MOESM1_ESM.doc]

**Intentional communication between wild bonnet macaques and humans**

**Supplementary Materials**

**Adwait Deshpande**^1*^, **Shreejata Gupta**^2#^ and **Anindya Sinha**^1,2,3,4,5^

^1^ Consciousness Studies Programme, National Institute of Advanced Studies, Bangalore, India

^2^ Animal Behaviour and Cognition Programme, National Institute of Advanced Studies, Bangalore, India

^3^ Primate Programme, Nature Conservation Foundation, Mysore, India

^4^ Centre for Neuroscience, Indian Institute of Science, Bangalore, India

^5^ Dhole’s Den Research Foundation, Bandipur National Park, India

**Corresponding author:*

**Adwait Deshpande**

Department of Comparative Cognition, Institute of Biology, University of Neuchatel Rue Emile-Argand 11 
CH-2000 Neuchâtel, Switzerland

Tel: +91- 8554005688

Email: adwait.deshpande2390@gmail.com / adwait.deshpande@unine.ch

**
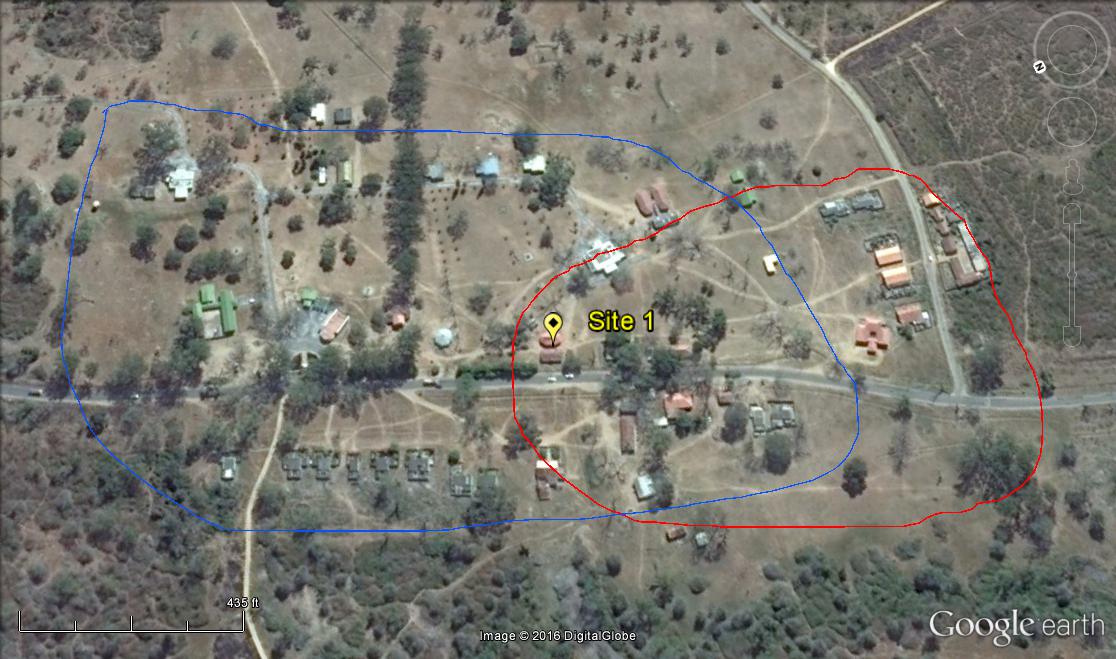
**

**Figure S1:** Google Earth image of the tourist reception area in the Bandipur National Park. Two polygons, in blue and red, show territories of two study troops of bonnet macaques C2 and C3 respectively (Image by Google and DigitalGlobe 2016)

**
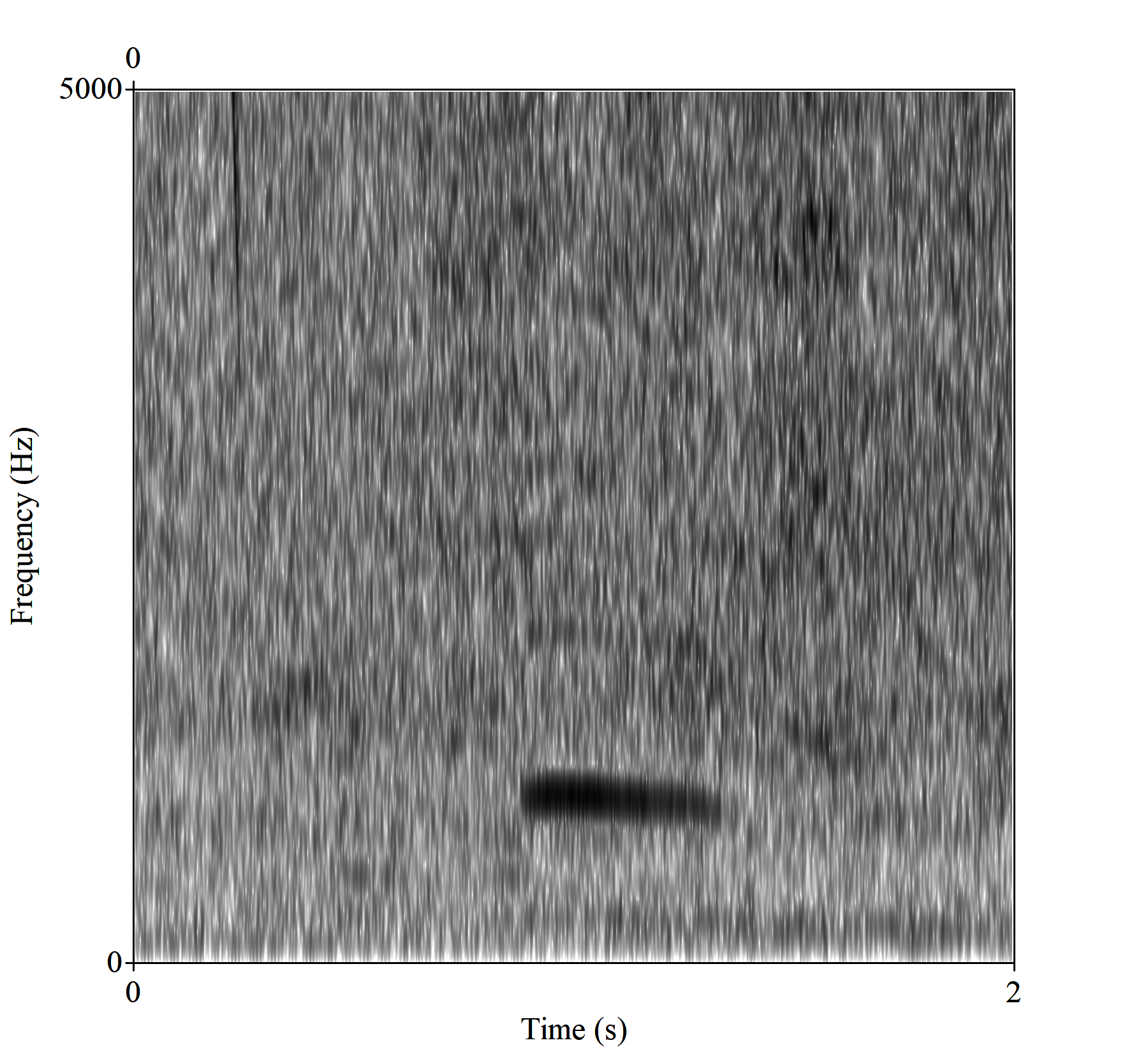
**

**Figure S2:** Spectrogram of a typical coo-call displayed by juvenile bonnet macaques during food requesting events. Spectrogram created in PRAAT (Hanning sine-squared window, view range of 0.0-5000 Hz, window length of 0.01 sec, dynamic range of 40.0 dB)


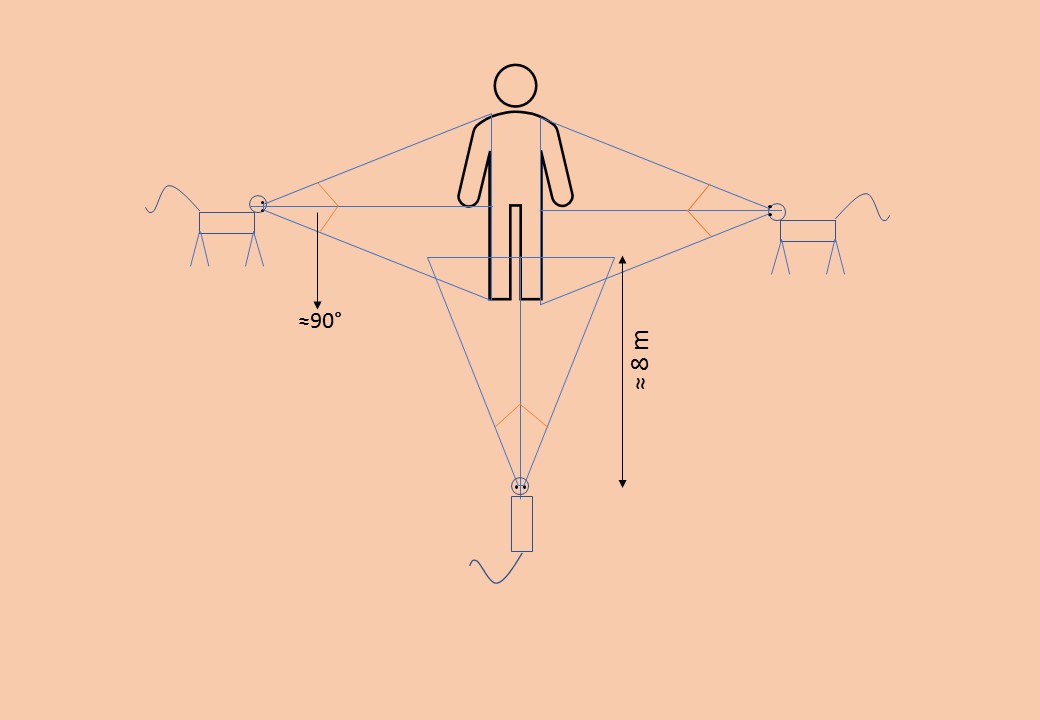


**Figure S3:** Graphic illustration of the monitoring behaviour of bonnet macaques during food-requesting bouts. The head of a subject macaque is completely orientated towards a human recipient, with the gazing towards the recipient being considered as visual monitoring of the recipient and/or the food item held by the recipient, spanning an angle of ≈ 90 deg either between the left and right or the top and bottom extremities of the human recipient, at a maximum distance of ≈ 8 m from the recipient


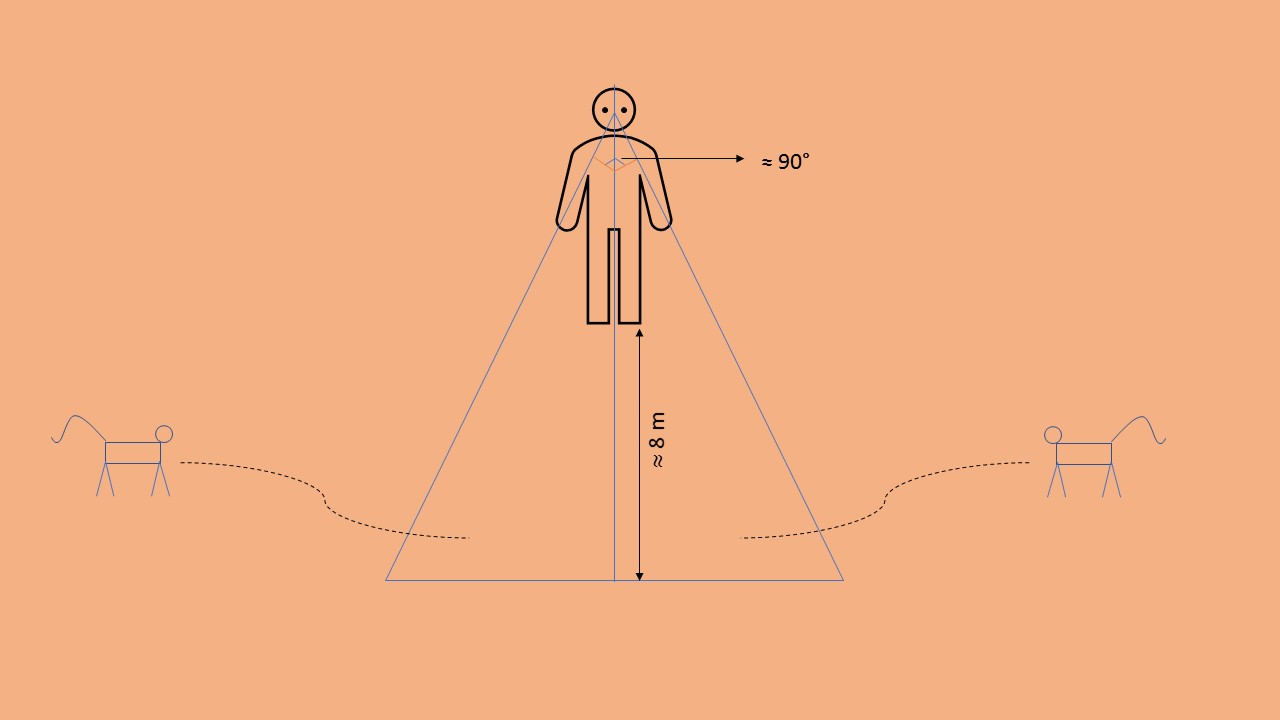


**Figure S4:** Graphic illustration of the orientation behaviour of bonnet macaques during food-requesting bouts. Orientation behaviour was defined as body movements or locomotor behaviour (change in position) displayed by a subject macaque that resulted in it positioning itself in the target’s line of vision, spanning an angle of ≈ 90 deg between the left and right extremities of the human recipient, at a maximum distance of ≈ 8 m from the recipient

**Supplementary Video Legends:**

**Video S1:** A natural food-requesting event, as obtained from natural observations

**Video S2:** An experimental trial, conducted during the study
